# Isoniazid-induced lichenoid eruption reaction

**DOI:** 10.11604/pamj.2023.45.162.40182

**Published:** 2023-08-15

**Authors:** Aishwarya Kishor Kedar, Pankaj Bandurao Wagh

**Affiliations:** 1 Department of Respiratory Medicine, Datta Meghe Institute of Higher Education and Research, Wardha, Maharashtra, India

**Keywords:** Pulmonary tuberculosis, erythematous, hyperpigmented, violaceous

## Image in medicine

A 50-year-old male patient presented with complaints of cough with mucoid expectoration and breathlessness for 6 months, along with multiple raised erythematous and hyperpigmented plaques all over the body for 1 month. He was vitally stable. He had a history of radiologically diagnosed pulmonary tuberculosis in October 2021 for which he took category 1 directly observed treatment short course [Tab Isoniazid 300 mg, Tab Rifampicin 450 mg, Tab Ethambutol 800 mg, Tab Pyrazinamide 750 mg] from October 2021 to March 2022. At the end of March 2022, he developed multiple raised erythematous, to begin with later violaceous, hyperpigmented itchy plaques all over the body. The patient's skin biopsy was taken from the back area and sent for histopathological examination. He was diagnosed to have an isoniazid-induced lichenoid eruption reaction. His systemic examination revealed reduced chest movements on the right side and the trachea was shifted to the right side [trail sign was positive] and had bilateral rhonchi and crepitations present all over the chest area. The patient was immediately admitted and his anti-tubercular medications were withheld. He was treated with low-dose oral glucocorticoid (Tab Prednisolone), glycerine and olive oil lotion, Tab Desloratadine along with intravenous antibiotics, nebulization, bronchodilator, and other supportive treatment. Later patient's sputum examination was tested which came out to be negative for acid-fast bacilli and he was discharged with symptomatic treatment after a reduction in the intensity of erythema and itching and was asked to follow up each month.

**Figure 1:**
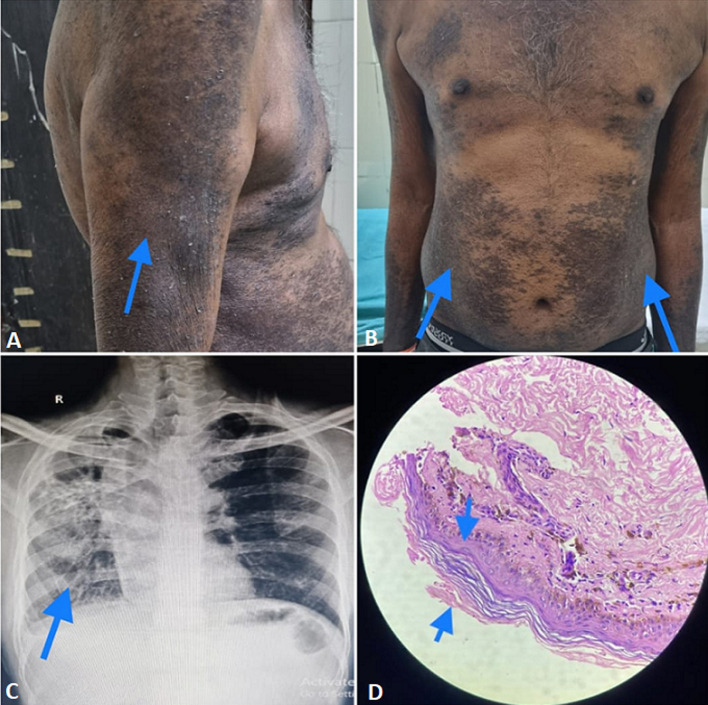
A, B) raised violaceous plaques on the trunk, abdomen and chest, and bilateral arms; C) chest X-ray posterior-anterior (PA) view revealing the trachea shifted to the right side and fibrotic changes in the right side of the lung; D) histopathological image revealing a dense band-like lymphocytic infiltrate in the dermo-epidermal junction, upper dermis, and perivascular region

